# The Displacements Study of Birch Veneer Layers from Composition of Plywood during Water Jet Cutting Using the Finite Element Method (FEA)

**DOI:** 10.3390/ma16124247

**Published:** 2023-06-08

**Authors:** Dorin-Ion Dumitrascu, Alexandru-Nicolae Rusu, Adela-Eliza Dumitrascu

**Affiliations:** 1 Department of Automotive and Transport Engineering, Transilvania University of Brasov, 1 Politehnicii, 500024 Brasov, Romania; d.dumitrascu@unitbv.ro; 2 Department of Manufacturing Engineering, Transilvania University of Brasov, 5 Mihai Viteazul, 500036 Brasov, Romania; alexandru.rusu@unitbv.ro

**Keywords:** layered board, water jet cutting, finite element method, displacements analysis

## Abstract

This paper presents a study of the deformations of the birch veneer layer of plywood composed of veneer sheets, each with a thickness of 1.4 mm. Displacements in the longitudinal and transverse directions were analyzed in each layer of veneer from the composition of the board. Cutting pressure was applied to the surface equal to the diameter of the water jet, located in the center of the laminated wood board. Finite element analysis (FEA) does not study the breaking of the material or its elastic deformation, but only what happens from a static point of view when maximum pressure acts on the board, which causes detachment of the veneer particles. The results of the finite element analysis indicate maximum values of 0.0012 mm in the longitudinal direction of the board located in the proximity of the application of the maximum force of the water jet. Additionally, in order to analyze the recorded differences between both longitudinal and transversal displacements, estimation of statistical parameters with 95% confidence intervals (CI) was applied. The comparative results indicate that the differences are not significant for the displacements under study.

## 1. Introduction

Due to the superior mechanical properties of layered wood boards (bending strength, modulus of bending elasticity, internal bond, etc.), the use of layered wood materials has actually increased in various applications.

Birch plywood has many advantages over other alternatives used for exteriors: resistance to moisture and chemicals, low cost, durable and light, safe and hygienic, reusable, and minimal impact on the environment. The layered material provides great homogeneity, which ensures a reduction in critical areas causing damage to the material under stress [1].

Birch plywood is a type of engineered wood product that is made by gluing together thin layers of birch wood veneers. The resulting plywood is strong, durable, and has a consistent appearance.

The water jet cutting usage described in this paper is considered an optimal solution for obtaining complex and precise cutting geometry compared with conventional technologies. Investigating the literature in the field showed that there are few studies on the application of the water jet cutting method to laminated plywood. Most of the studies have analyzed the hardwood cutting process. However, studies on the use of abrasive water jet cutting (AWJ) in the processing of composite materials or wood-based laminated materials are diverse [2,3,4,5,6,7,8]. These studies focused mainly on the assessment of the optimal process parameters for abrasive water jet cutting [9,10,11]. It can be highlighted that, in particular, the emphasis was on the investigation and optimization of abrasive water jet cutting parameters in order to improve the cutting quality of composite materials. Identification of the optimal parameters that provide the best machining quality and the effects of parameters such as kerf taper and surface roughness was studied [12,13].

The Taguchi method (Design of Experiments) was widely applied as the experimental approach. Shanmugam and Masood [14] developed a predictive model correlating the kerf taper angle with the process variables. Using an analytical and graphical optimization technique based on analysis of variance (ANOVA) [15], the optimum cutting parameters were identified, and the effects of the combined cutting parameters were studied [12]. Additionally, the ANOVA method has been used in the optimization of the jet-cutting process by many authors in order to increase the dimensional accuracy and improve the quality of the obtained surfaces.

Regarding the abrasive water jet for cutting solid wood, the experimental research conducted by [10] aimed to determine the surface roughness of three types of wood widely used in the woodwork industry and in furniture-decorating applications (Scotch pine—*Pinus sylvestris Lipsky*, Eastern beech—*Fagus orientalis Lipsky*, and sessile oak—*Quercus petraea Lieble*), setting different processing parameters and with different thicknesses (9 mm, 18 mm, 36 mm, and 54 mm) in tangential and radial directions. In order to determine the influence of the variables, the following parameters were analyzed: feed speeds (50 mm/min, 100 mm/min, and 200 mm/min), abrasive flow rates (200 g/min, 300 g/min, and 450 g/min), wood thickness, cutting direction, and two different cutting liquid pressures (300 MPa and 380 MPa). The experimental results showed that the most important parameters are feed speed and wood thickness, which influence roughness. Cutting direction and liquid pressure had a minimum influence on the Ra and Rz roughness.

The finite element method (FEA) is one of the most used simulation methods for the analysis of wood materials, with accurate results [16,17,18,19]. The application of FEM to bending wood was conducted to determine the stress behavior and the impact of different compositions of layered materials on individual layers. The results indicated that the deflection values decreased as material thickness increased [1]. Water jet cutting is a manufacturing process that uses a high-pressure jet of water or a mixture of water and an abrasive material. Pure water jets are used for softer materials such as rubber, plastics, or wood. The abrasive material is added to the water jet to cut hard materials such as metal, glass, or stone. In the case of plywood, due to its mechanical properties, a high-pressure stream of water is typically combined with an abrasive material to increase the cutting power of the water and to obtain a smooth surface. The abrasive material is added to the water stream before it exits the orifice. The stream of the abrasive particles (about 50 μm grit size with sharp irregular edges) is introduced into the water jet cutting machine. In this way, the pressure of the water jet machine is transferred to the abrasive particles. The water jet machine moves the water jet stream along a pre-programmed path, guided by IntelliMAX software, ver. 24.0. As the water jet stream hits the surface of the plywood, it begins to cut into the material, creating a narrow kerf. The water jet can cut through the plywood by creating a series of small fissures or cracks along the path of the cut. The high-pressure water stream and abrasive material work together to widen the cracks, creating a wider kerf and separating the material into two pieces. The cutting speed of the water jet can be adjusted to match the thickness and hardness of the plywood. Thicker and harder plywood may require a slower cutting speed to ensure that the water jet can penetrate through the material.

One of the main advantages of water jet cutting compared with traditional technologies in the field of wood cutting is the precise cutting method that can produce complex shapes and patterns with very tight tolerance. It is also a relatively cold cutting process, which means that it does not generate a lot of heat or thermal stress on the material being cut. This reduces the risk of thermal damage to the surrounding medium, such as warping or discoloration. In this regard, the water jet cutting generates smooth but free surfaces. In this manner, secondary finishing is not needed. This makes it an ideal cutting method for materials such as plywood that can be sensitive to heat or warping. Additionally, the process of cutting plywood with a water jet can have an impact on the surrounding medium. However, the impact is typically minimal compared with other cutting methods such as laser cutting or plasma cutting.

Additionally, the water jet stream is contained within a cutting table or enclosure, which helps to prevent the dispersion of water and abrasive material into the surrounding environment. This reduces the risk of contamination and damage to nearby surfaces.

However, it is important to note that the water and abrasive material used in the cutting process can still have some impact on the immediate area. For example, the water and abrasive material can cause some erosion of the cutting table or enclosure over time, and the noise generated by the water jet machine can be relatively loud.

It is well known that water is not a very simple factor when working with wood veneer. The problem of cutting with a water jet arises when water comes into contact with the layered wood plywood, both during cutting and immersion of the board, which negatively influences the properties of the veneer layers. In this sense, the current study aims to apply the finite element method (FEA), which will allow tracking of the deformation of the laminated wood board in the direction of the water pressure. For the proposed study, the longitudinal and transverse displacements obtained for each individual veneer layer will be analyzed. This study is only valid when the water jet first hits the first layer of veneer. After the first layer of veneer is broken, the maximum displacement moves to the next layer of the veneer, and the process is repeated until the laminated wood board is completely broken.

## 2. Materials and Methods

Considering the laminated plywood applications, there are different selection criteria, namely, structural wood design (EN 1995-1-1) [20], plywood performance characteristics (EN 13986) [21], requirements for plywood adhesives (EN 314-2) [22], and plywood durability depending on the exploitation class (EN 335) [23].

Taking into account the usage fields, laminated plywood is divided into three classes (EN 314-2) [22]:

Class 1—interior applications without moisture risk. Plywood humidity must not exceed 12%, and ambient humidity must not be less than 65% at 20 °C;

Class 2—external applications, but protected. Plywood humidity must not exceed 20%, and the ambient humidity must be between 65% and 85% at 20 °C;

Class 3—fully exposed exterior applications. Plywood humidity exceeds 20%.

In accordance with EN 635-2 European norms [24], the most common classes of birch plywood are as follows:

- B/BB: This is the most common grade of birch plywood. It has a B-grade face and back veneer, and a core made up of several layers of birch veneer. This grade is suitable for a wide range of applications, including furniture, cabinetry, and interior decoration.
- BB/BB: This grade of birch plywood has a BB-grade face and back veneer, and is made up of several layers of birch veneer. It is similar to the B/BB-grade plywood, but with a higher-quality face and back veneer. This grade is suitable for applications where appearance is important, such as furniture and decorative panels.
- BB/CD: This grade of birch plywood has a BB-grade face and a C-grade back veneer. The core is made up of several layers of birch veneer. This grade is suitable for applications where appearance is important on one side only, such as wall panels or cabinet backs.
- CD/DD: This grade of birch plywood has a C-grade face and back veneer, and a core made up of several layers of birch veneer. This grade is suitable for applications where appearance is not important, such as subfloors or roofing.

Supplementary, additional classes of birch plywood are available from specific manufacturers, and specific plywood characteristics may vary depending on the manufacturing process and intended use.

For Romania, the standard requirements are briefly presented in Table 1.

Quality classes of laminated plywood refer to:

B—The highest quality, sanded, without discoloration and external defects; can be painted or varnished;

BB—Good-quality, sanded, small healthy knots, and veneer joints are accepted; can be varnished and painted;

CD—Standard class of plywood quality, sanded, accepts veneer joints, inserts, and corrected defects; presents more knots than the BB class;

C—Low-quality class presents different colorations on the board, knots, and small missing veneer holes;

D—Low-quality class presents different colorations on the board, knots, and small missing veneer holes.

In this study, outdoor birch plywood with the following characteristics was used: 720–780 kg/m^3^ specific weight, 2500 mm × 1250 mm standard size, and 15 mm thickness.

Each layer contains two axes: longitudinal and transversal. In the FEA simulation, orthotropic mechanical properties for the birch veneer were used (Table 2). The properties of the analyzed plywood are provided by the Odek supplier. These mechanical properties provide valuable information about birch’s behavior under different types of stress, and its structural performance analysis can be used for various applications. On the layered wood board, a cutting pressure equal to the water jet diameter located in the center of the board was applied. Since the rupture of the material or its elastic deformation is not studied, in the current analysis, only the area of material elastic deformation was considered. Additionally, for simulation with finite elements, the adhesive layer for gluing veneer boards was not considered.

**Figure 1:**
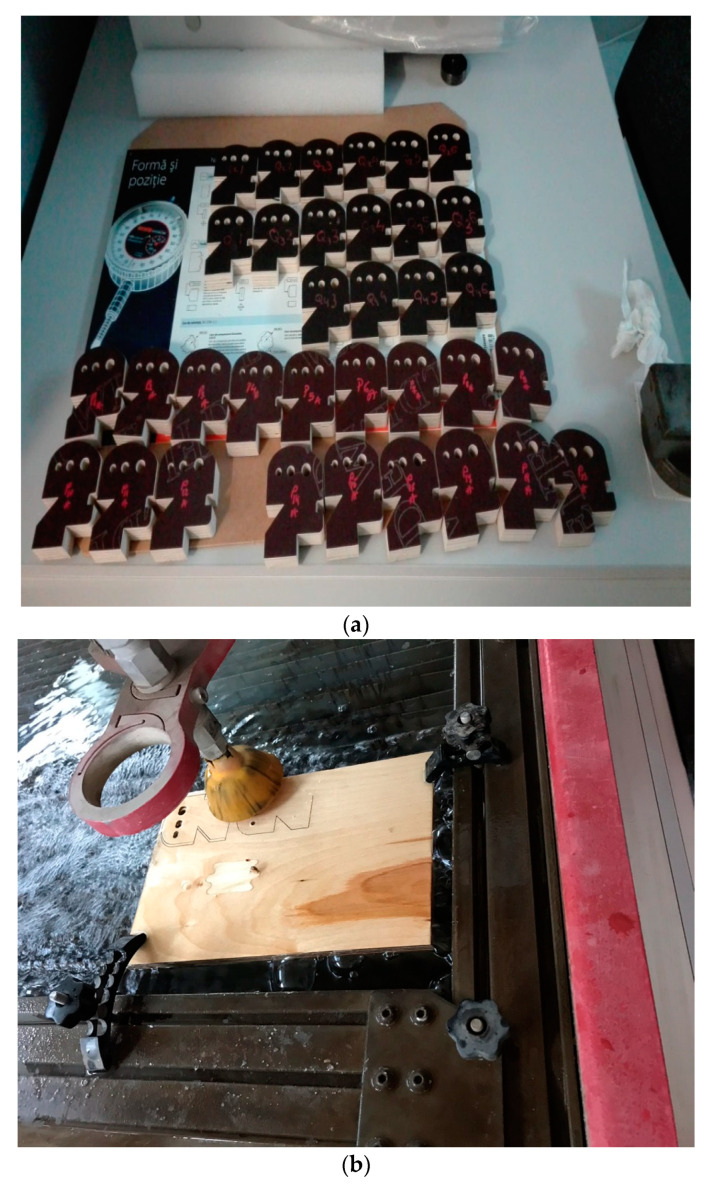
Cutting of layered wood samples in AWJ system: (**a**) layered wood samples; (**b**) cutting process.

To cut layered wood samples with different parameters, the abrasive water jet cutting machine equipped with numerical control was used. The abrasive material is alluvial garnet sand, the place of provenience is Constanta, Romania, the grain size 7–8 Mohs, the specific gravity is 3.9–4.1 g/cm^3^, and the bulk density is 1.8–1.9 g/cm^3^, with a sub angular grain shape, and a red-green color. The main parameters of the abrasive water jet cutting machine are presented in Table 3. Additionally, the samples obtained through the cutting processing with abrasive water jets are presented in Figure 1.

### 2.1. Geometric Modeling of the Composite Board and Material Properties

In order to analyze the deformations that occur due to water jet pressure, analysis of the finite elements of laminated wooden board made of birch veneer for the exterior with standard dimensions of 2500 mm × 1250 mm was considered. The laminated wood board had a thickness of 15 mm and consisted of 11 veneer layers, each layer having 1.4 mm thickness, oriented at 90 degrees to each other, one longitudinal and the other in a transversal direction, and providing additional resistance to the layered board. To glue the veneer boards, the Dynea brand adhesive with moisture resistance of BFU 100 was used.

### 2.2. Statistical Analysis

Comparative analyses of longitudinal and transversal displacements were statistically processed using Minitab 17 software (Minitab LLC, State College, PA, USA). To validate the statistical distribution, both the goodness-of-fit test expressed by Pearson’s correlation coefficient and the least squares estimation method were applied.

The most popular methods are least squares (LS) and maximum likelihood estimation (MLE), which are both implemented for parametric estimation [25,26,27].

Given a continuous random variable *X*, the functions are given by [25,26,27]:

- *f(x)* is the probability density function (pdf);
- *F(x)* is the cumulative density function (cdf).

If *X* is a continuous random variable, then the probability density function of *X* is *f(x)*, such that for two numbers, *a* and *b* with *a ≤ b*, we achieve:

(1) $$P\left(a\leq X\leq x\right)=\int_{0}^{x}f\left(x\right)dx.$$

The cumulative distribution function is a *F(x)* function of a random variable *X,* and it is defined for a number *x* by:

(2) $$F\left(x\right)=P\left(X\leq x\right)=\int_{0,-\infty }^{x}f\left(x\right)dx.$$

Specific to Weibull distribution, the main indices are given by the following equations [25,26,27]:

(3) $$f\left(x_{i}\right)=\frac{\beta }{\eta }\cdot {\left(\frac{x_{i}-\gamma }{\eta }\right)}^{\beta -1}\cdot e^{-{\left(\frac{x_{i}-\gamma }{\eta }\right)}^{\beta }},$$

(4) $$F\left(x_{i}\right)=1-e^{-{\left(\frac{x_{i}-\gamma }{\eta }\right)}^{\beta }},$$

(5) $$R\left(x_{i}\right)=e^{-{\left(\frac{x_{i}-\gamma }{\eta }\right)}^{\beta }},$$

(6) $$h\left(x_{i}\right)={\left(\frac{x_{i}-\gamma }{\eta }\right)}^{\beta -1},$$

where *β* is the shape parameter, *η* is the scale parameter and *γ* is the location parameter.

Based on the FEA estimated displacements, in order to analyze the goodness of fit, the most frequent distributions were chosen. For parametric distribution analysis, Weibull, lognormal, exponential, and normal probability distributions were considered. In order to highlight the difference between both deformations in the specific areas of the veneer layers of laminated wood board, the descriptive statistics were estimated. With a 95% confidence interval, inferential analysis of distributions, probability plots, and hazard rate were applied.

### 2.3. Description of the Finite Element Model and Application of Boundary Conditions

**Figure 2:**
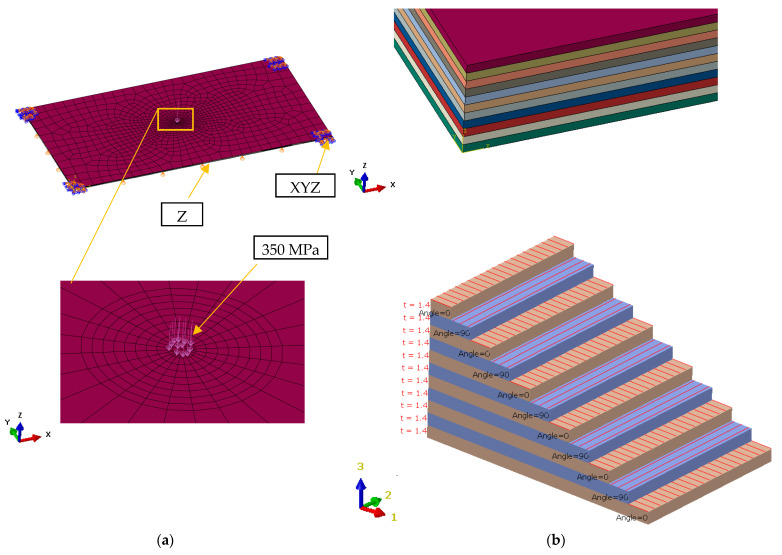
The finite element model for the layered board: (**a**) boundary conditions; (**b**) orientation of the veneer corresponding to each layer of the board component.

Figure 2 shows the finite element model for the layered board using Abaqus CAE software, ver. 6.14. The layered board is modeled according to 3D finite elements and hexahedron type, and is assigned material properties and fiber orientation for each veneer layer. The number of elements is 16,962 and the number of nodes is 18,972. Mesh size is variable, the minimum size factor is 0.1, and the maximum size is 100 for important zones. The connection between layers in structure composition is made through common nodes between finite elements.

Boundary conditions for the finite element model are set in order to obtain results closer as possible to reality.

To simulate the pressure applied to the board, 350 MPa water jet pressure was used. The pressing surface is equal to the area of the circle given by the diameter of the water jet, which is Φ 0.28 mm. During the cutting process, the board rests on the cutting table and is fixed at four points. The same behavior was reproduced on the board corners in the simulation. In the finite elements model, the board is fixed in all directions on the corners and on the opposite side of applied pressure, the movement in the normal direction is also blocked.

## 3. Results

### 3.1. The Results Obtained from the Finite Element Analysis

Using the finite element method, it was observed how much the layered board was deformed under water pressure.

**Figure 3:**
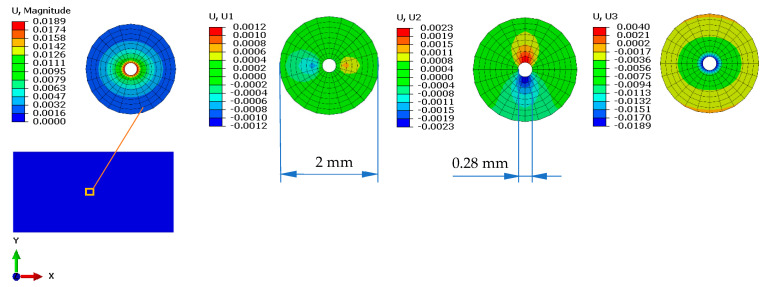
Displacement distribution—view from the direction of force application.

Figure 3 shows the maximum displacements obtained on the layered board in the three directions, as well as their magnitude. As can be seen, the longitudinal and transversal deformation values are very small, having a local effect with a radius of approximately 1 mm. Even if the deformation in the direction of pressing force is the largest, it was not analyzed because, in reality, the material in this area breaks during the cutting process.

For the proposed study, the displacements recorded in longitudinal and transversal directions obtained for each individual layer of veneer were analyzed.

### 3.2. The Displacements of Veneer Layers in the Longitudinal Direction

**Figure 4:**
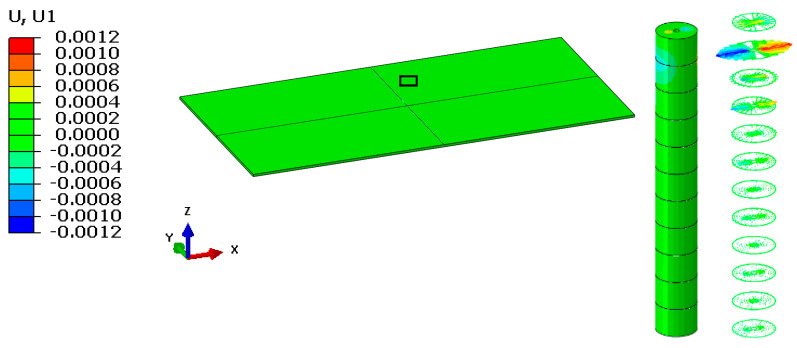
Deformation of the layered board in the longitudinal direction.

**Figure 5:**
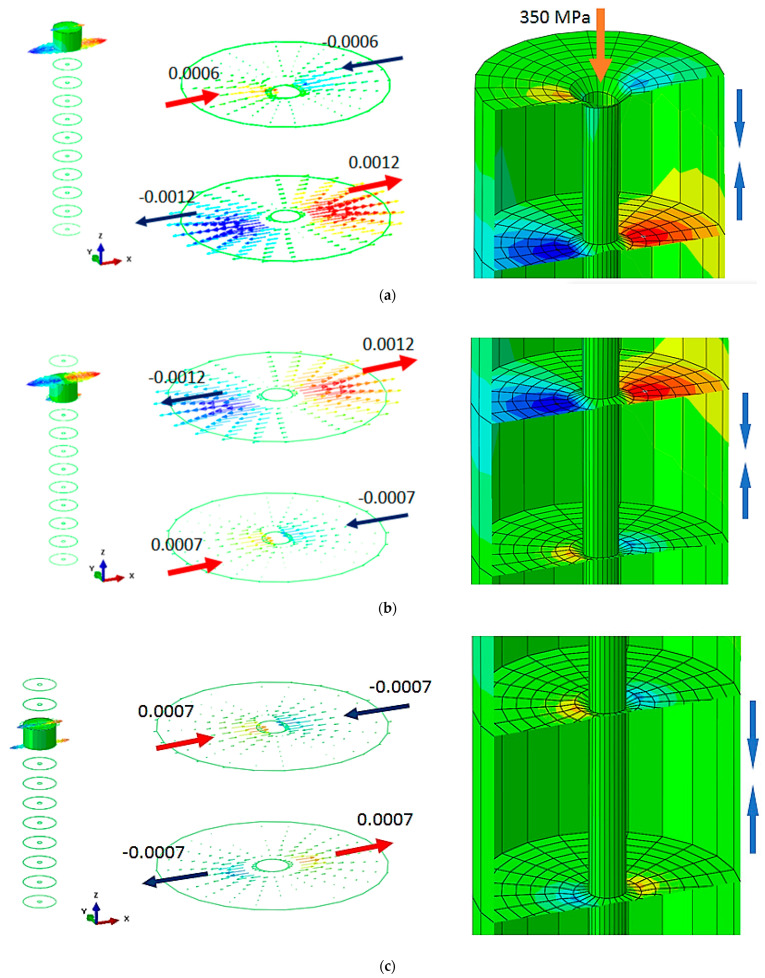

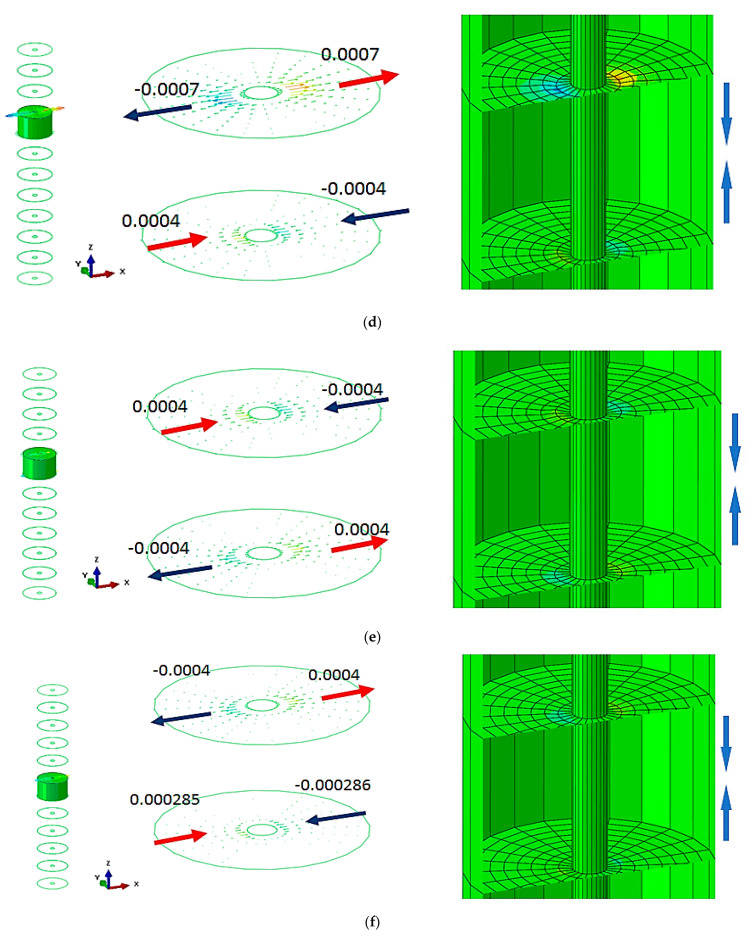

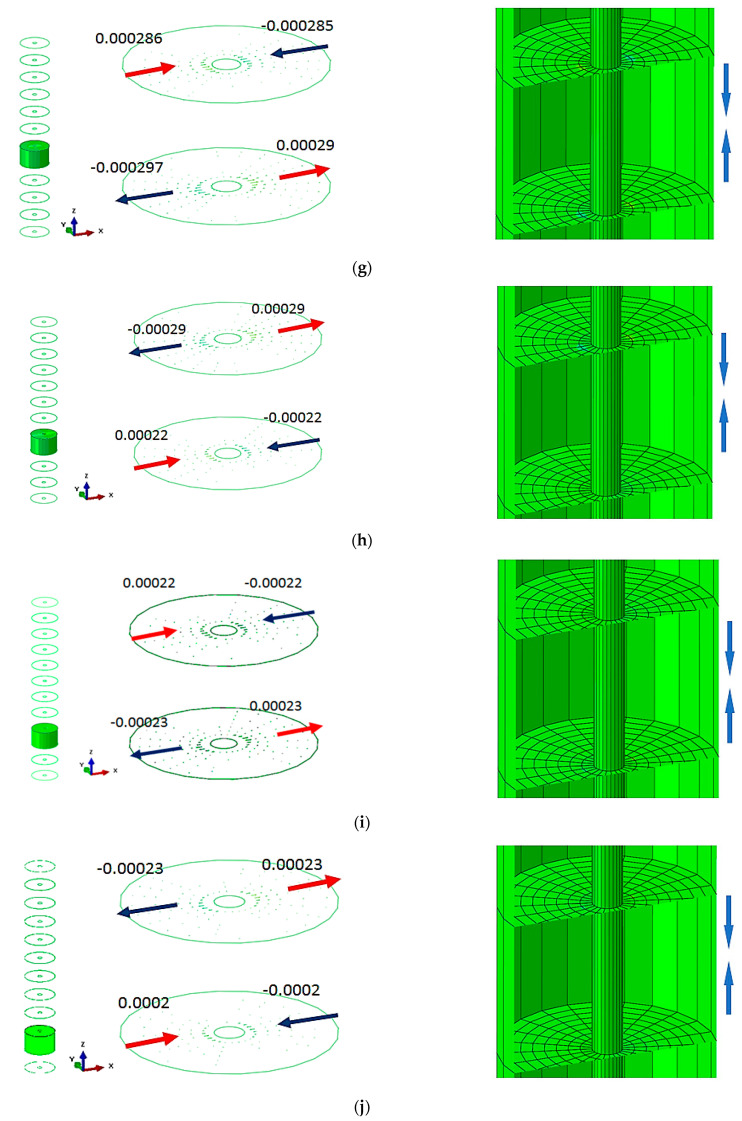

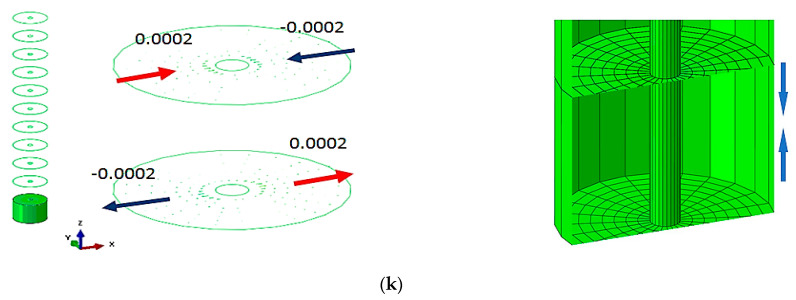
Deformation in the longitudinal direction in the common areas of the veneer layers: (**a**) specific displacement of layer 11; (**b**) specific displacement of layer 10; (**c**) specific displacement of layer 9; (**d**) specific displacement of layer 8; (**e**) specific displacement of layer 7; (**f**) specific displacement of layer 6; (**g**) specific displacement of layer 5; (**h**) specific displacement of layer 4; (**i**) specific displacement of layer 3; (**j**) specific displacement of layer 2; (**k**) specific displacement of layer 1.

As can be seen in Figure 4, the maximum value of displacement in the longitudinal direction of the board is 0.0012 mm when placed between 10 and 11 veneer layers in the vicinity of the maximum force of the water jet application. Since the layered board is large enough, the resultant displacements had a very small local radial effect with a radius of approximately 1 mm. This effect was observed for all the veneer layers in the composition of the board. The values of the longitudinal displacements in the common interface formed by the pairs of veneer layers are presented in Figure 5. It can be observed that displacements decrease depending on the thickness of layered board at intervals of two veneer layers.

It must be taken into account that this effect appears only when the water jet hits the first veneer layer. After the first veneer layer (11) was broken, the maximum displacement of 0.0012 mm was transmitted to the next veneer layer (layer 10), and the process was repeated until the layered board was completely broken. Essentially, the breaking effect took place gradually, one layer at a time, until the board was completely covered.

The vertical displacement of the material follows the principle of action and reaction. If the first layer is acted upon by the force of the water jet, the veneer layer moves in the direction of the force; however, the next layer reacts in the opposite direction of the application of the force, and the displacement has the same direction as the force. Displacement values in the vertical direction are not relevant in this situation, but the deformation mode between 10 and 11 veneer layers is important considering the direction of applied force.

**Figure 6:**
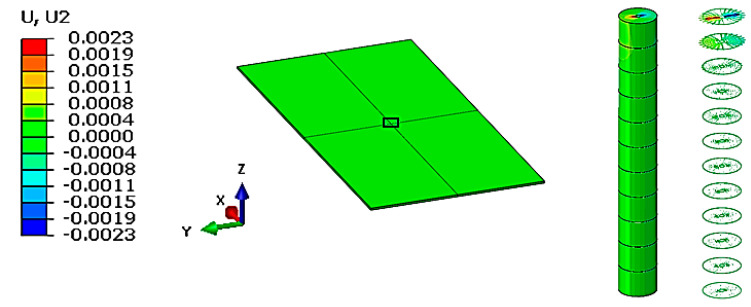
The deformation of the layered board is in a longitudinal direction.

Minimum and maximum displacement values in the longitudinal direction specific to common areas in the veneer layers, as well as the deformation mode in the pressure application area, are presented in Figure 6.

### 3.3. The Displacements of the Veneer Layers in the Transverse Direction

The maximum displacement in the transverse direction of the board is 0.002 mm, being positioned on the first veneer layer, where the water pressure is applied for board cutting. Since the board size in the transverse direction is smaller compared with the longitudinal one, the board is more rigid and larger deformations appear in the area of applied force; there was an accentuated decrease with the board thickness. In this case, too, the displacement effect is noted on an area of approximately 1 mm.

**Figure 7:**
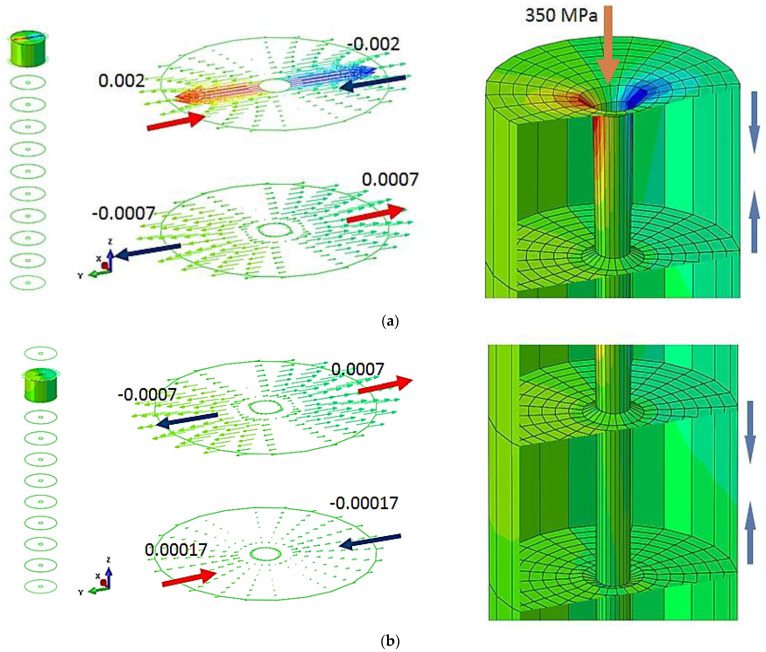

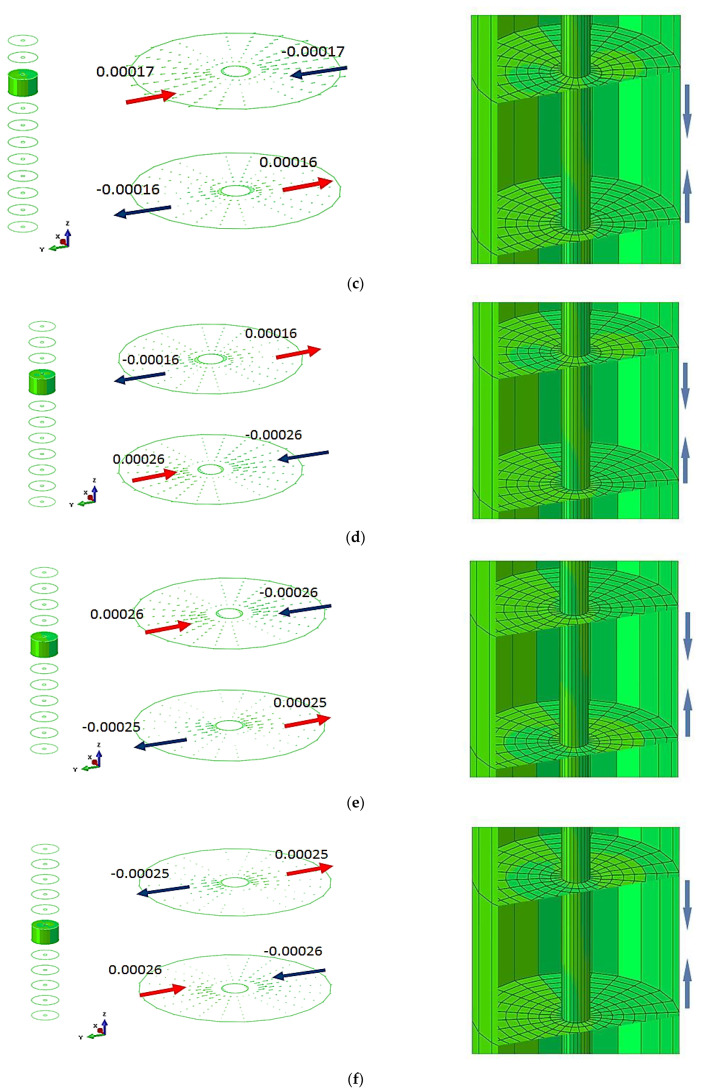

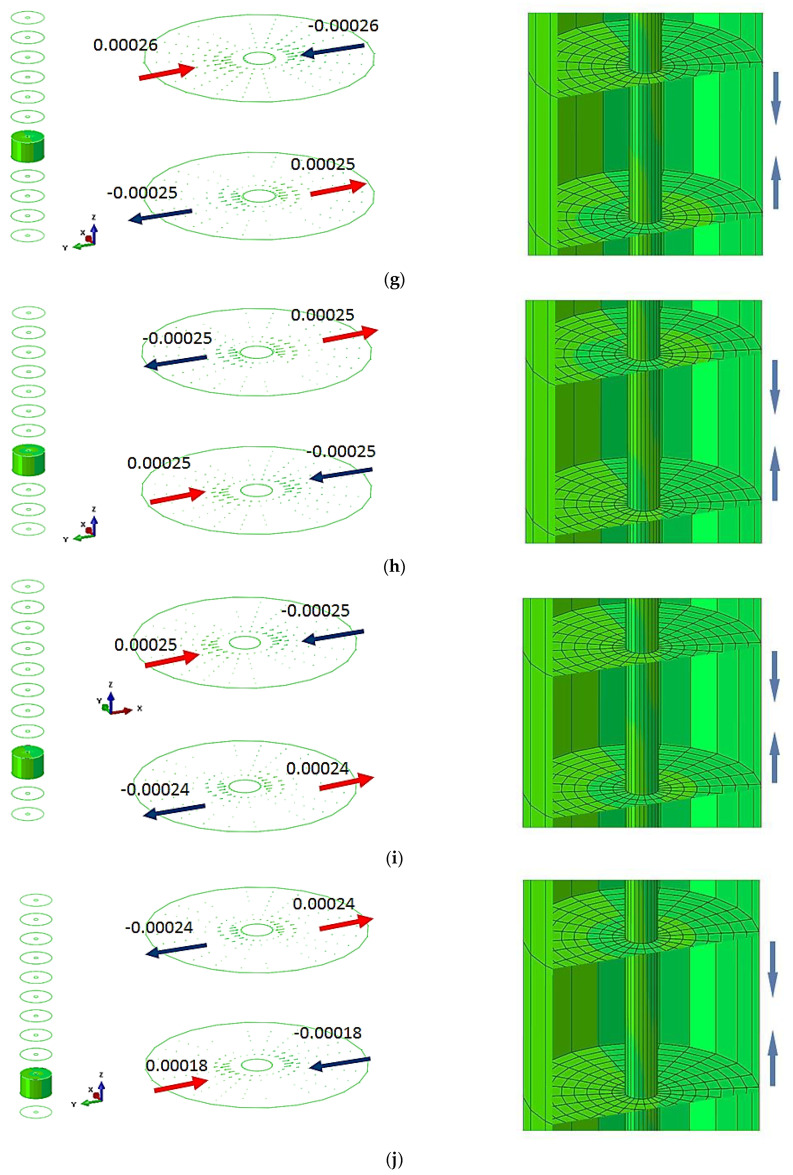

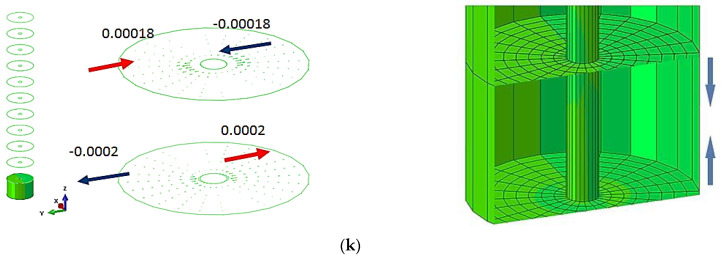
Deformation in the transverse direction in the common areas of the veneer layers.: (**a**) specific displacement of layer 11; (**b**) specific displacement of layer 10; (**c**) specific displacement of layer 9; (**d**) specific displacement of layer 8; (**e**) specific displacement of layer 7; (**f**) specific displacement of layer 6; (**g**) specific displacement of layer 5; (**h**) specific displacement of layer 4; (**i**) specific displacement of layer 3; (**j**) specific displacement of layer 2; (**k**) specific displacement of layer 1.

The deformation mode in the pressure application area and transverse displacement values for each common interface formed by pairs of veneer layer are shown in Figure 7.

### 3.4. Statistical Analysis of the Displacement of Veneer Layers

To emphasize the significant differences between the displacements obtained via finite element analysis, the inferential analysis consists of evaluation of veneer layer displacements in the longitudinal and transversal directions by estimation of the statistical parameters, the probability density function and hazard rate analysis with a 95% confidence interval.

**Figure 8:**
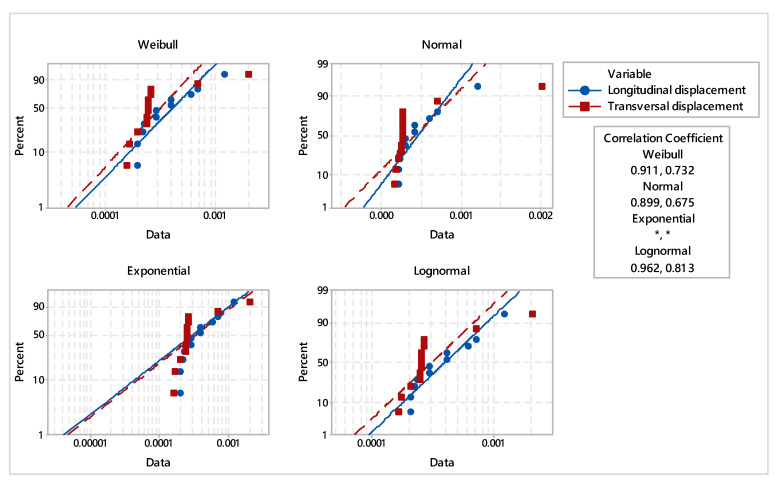
Probability plot of analyzed displacement.

**Figure 9:**
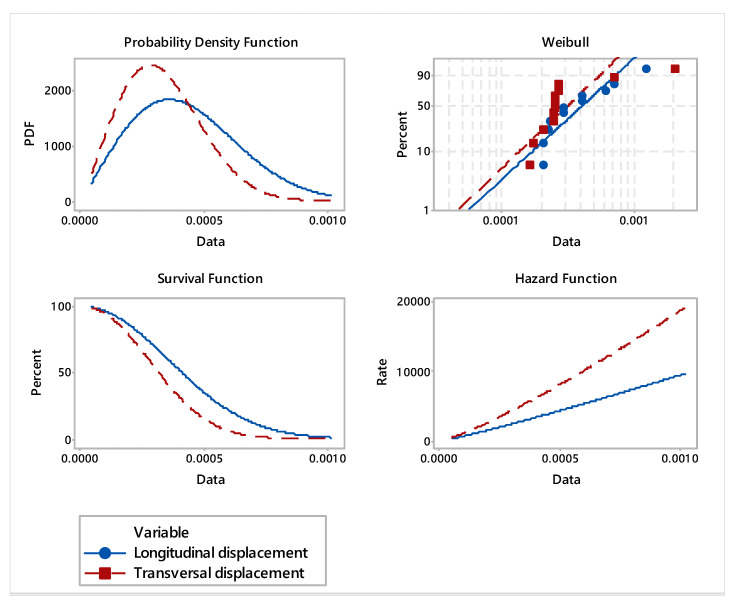
Distribution overview plot for longitudinal and transversal displacement.

The probability plots and probability density distribution of the fitted distributions of the specific displacements are shown in Figure 8 and Figure 9. The results of the goodness-of-fit test are expressed via the Pearson correlation coefficient, applying the least squares estimation method. It was found that the displacements complied with Weibull distribution, where the values of the correlation coefficient were 0.911 for longitudinal displacement and 0.732 for transversal displacement, respectively.

In accordance with the analyzed distributions, the estimated statistical parameters values of veneer layers displacement are synthetically presented in Table 4, Table 5 and Table 6. The transversal displacement indicates a higher median value (median = 0.00033) compared with longitudinal displacement (median = 0.00021). However, the values of the estimated means and medians of the displacements under study are comparable. Although the maximum displacement is recorded in the transverse direction of the layered wood board, it can be also noticed a large variation of data observed for longitudinal direction. Additionally, specific to the first two layers of veneer, in the area of application of the maximum force of the water jet, the probability density function records a major deformation. Additionally, the hazard rate reveals a more accentuated deformation of veneer layers on transversal direction. This is highlighted by the fact that the layered board has different stiffnesses in both directions.

The overall comparative analysis of the analyzed displacements indicates that transversal displacement is superior compared with longitudinal displacement. However, the probability plots, the probability density function and estimated statistical parameters reveal that the difference is not significant for analyzed types of displacements.

## 4. Discussion

The advantages of the application of nonconventional technology of abrasive water jet cutting it is reflected by high accuracy, high productivity, high flexibility, low dust particles, without heat generation and being environmentally friendly. In these respects, nonconventional processes such as abrasive water jet (AWJ) machining, laser cutting, electric discharge machining (EDM), etc., represent viable alternatives.

The cost of cutting plywood with an abrasive water jet system can vary depending on a number of factors, such as the thickness of plywood, the complexity of the cut and the abrasive material type used. Additionally, water jet cutting can be a relatively fast and efficient cutting method, especially when compared with manual cutting processes such as sawing or drilling. This can help to reduce labor costs and increase productivity.

One advantage of water jet cutting is that it can cut through plywood without generating a lot of waste or scrap material. This is because the water jet can make very precise cuts, allowing for maximum use of the material.

While water jet cutting machines can be expensive to purchase and maintenance, they can provide a high return on investment over time, especially in industries where precision cutting and high productivity are important.

Experimental studies and simulated research, especially the application of FEA analysis regarding the cutting of birch plywood using AWJ technology, are quite reduced (limited). Frequently, studies are available that highlight the application of AWJ for cutting solid wood, namely hardwood and softwood material.

The FEA results indicate that the deformation of veneer layers are in proximity with the application of the maximum force of the water jet. In this regard, the water jet pressure is the main cutting parameter that can influence the deformation of the veneer layers of the layered board. In this sense, the most important cutting parameter that can influence the deformation of veneer layers is the water jet pressure. Additionally, it is very important to underline the effect of stress accumulation into the entire structure, which can influence the quality, strength and performance of the plywood. In this context, these types of effects and similar results for the distribution of bending stresses into plywood layers were detailed by [9]. He found that stress distribution within the range of elastic deformation is linear in layers with an identical grain orientation. Additionally, distribution varies between veneers depending on their mechanical properties, and the stresses change stepwise depending on the values of deformation and the modulus of elasticity for each individual veneer.

In order to determine the impacts of various compositions of layered materials and the thickness of individual layers, the analysis of simulated stresses associated with the bending of wood using the Finite Element Method was developed as shown in [1]. The results indicate that with the increase in thickness, the bendability coefficient value decreases and bending force increases.

## 5. Conclusions

- The aim of the study was to evaluate the viability of applying of this cutting process to such type of material. In this regard, the FEA analysis was validated through experimental tests.
- Maximum displacement in the longitudinal direction of the board is 0.0012 mm and was located between 10 and 11 veneer layers, being generated by water jet pressure.
- The maximum displacement in the transversal direction of the board is 0.002 mm and is positioned on the first layer, where the water pressure is also applied for cutting the board.
- Since the layered board is large enough, the resulting displacements have a very small local radial effect in a radius of approximately 1 mm.
- Displacements decrease depending on the thickness of the layered board. In the longitudinal direction, they decrease more gradually, and in the transverse direction they register a sudden decrease due to the different stiffness of the board in the two directions (Figure 9).
- The breaking effect takes place step by step, one layer at a time, until the board is completely cut.
- The vertical displacement of the material follows the principle of action and reaction. If the first layer is acted upon with the force given by the water jet, the veneer layer moves in the direction of the force, and the next layer reacts in the opposite direction of the force application, and the movement has the same direction as force.
- The values of displacements in the vertical direction are not relevant. In particular, the veneer deformation pattern can be noticed between 10 and 11 layers in the direction of force application.
- Test results indicate no statistically significant differences for longitudinal and transversal displacements.

## Figures and Tables

**Table 1:** Quality classes for birch plywood.

Quality Class *	Requirements
Aesthetic veneer B/B	Both sides without knots
Aesthetic veneer B/C	One side without knots and one side with more than 3 knots
Aesthetic veneer C/C	Both sides with more than 3 knots
Aesthetic veneer C/D	One side with more than 3 knots and the other side with many knots that can fall off
Aesthetic veneer I	Both sides without knots
Aesthetic veneer II	One side without knots and one side with more than 3 knots
Aesthetic veneer III	Both sides with more than 3 knots
Aesthetic veneer IV	One side with more than 3 knots and the other side with many knots that can fall off

* EN 635-2.

**Table 2:** Mechanical properties for birch.

Nr. Crt	Property	Units	Value
1	ρ	kg/cm^3^	913
2	E_L_ (E_1_)	MPa	15,290
3	E_T_ (E_2_)	MPa	764.5
4	E_R_ (E_3_)	MPa	1192.62
5	µ_LR_ (ν_13_)	-	0.426
6	µ_LT_ (ν_12_)	-	0.451
7	µ_RT_ (ν_32_)	-	0.697
8	µ_TR_ (ν_23_)	-	0.426
9	µ_RL_ (ν_31_)	-	0.043
10	µ_TL_ (ν_21_)	-	0.024
11	G_LR_ (G_13_)	MPa	1131.46
12	G_LT_ (G_12_)	MPa	1039.72
13	G_RT_ (G_32_)	MPa	259.93
14	Tension strength parallel to grain	MPa	6.3
15	Tension strength perpendicular to grain	MPa	6.3
16	Compression strength parallel to grain	MPa	56.3
17	Compression strength perpendicular to grain	MPa	6.7
18	Shear strength parallel to grain	MPa	13

**Table 3:** Machining parameters in AWJ cutting.

Cutting Parameter	Value
Wood thickness	15 mm
Wood cutting direction	Tangential, radial
Feed speed	759–3381 mm/min
Liquid pressure	137.8–344.7 MPa
AWJ nozzle diameter	0.2794 mm
AWJ nozzle length	80 mm
Abrasive flow rate	0.3402 Kg/min
Total time spent cutting	0.62 s
Estimated abrasive needed	0.05 Kg
Length of cutting	172.406 mm
Mixing tube diameter	0.8382 mm

**Table 4:** Results of longitudinal displacement analysis.

Distribution	Correlation Coefficient	Mean	Standard Error	95% CI	
				Lower	Upper
Weibull	0.911	0.0004345	0.0000645	0.0003249	0.0005812
Normal	0.899	0.0004525	0.0000843	0.0002872	0.0006178
Exponential	-	0.0004262	0.0001194	0.0002461	0.0007381
Lognormal	0.962	0.0004604	0.0000906	0.0003130	0.0006771

**Table 5:** Results of transversal displacement analysis.

Distribution	Correlation Coefficient	Mean	Standard Error	95% Confidence Interval	
				Lower	Upper
Weibull	0.732	0.0003342	0.0000558	0.0002410	0.0004635
Normal	0.675	0.0004150	0.0001092	0.0002010	0.0006290
Exponential	-	0.0004752	0.0001468	0.0002594	0.0008706
Lognormal	0.813	0.0003590	0.0000686	0.0002468	0.0005222

**Table 6:** Descriptive statistics of analyzed displacements.

Parameter	Longitudinal Displacement [mm]	Transversal Displacement [mm]
Median	0.0002162	0.0003342
Standard deviation	0.0003004	0.0001604
Mean	0.0004345	0.0003342
IQR	0.0003006	0.0002236
Shape parameter	2.1130100	2.1995300
Scale parameter	0.0004906	0.0003773

## Data Availability

Not applicable.

## Nomenclature

*ρ*:	Mass density;
*E_L_*:	Young’s modulus in the longitudinal direction;
*E_T_*:	Young’s modulus in the transverse direction;
*E_R_*:	Young’s modulus in the radial direction;
*μ_LR_*:	Poisson’s ratio for longitudinal–radial;
*μ_LT_*:	Poisson’s ratio for longitudinal–transverse;
*μ_RT_*:	Poisson’s ratio for radial–transverse;
*μ_TR_*:	Poisson’s ratio for transverse–radial;
*μ_RL_*:	Poisson’s ratio for radial–longitudinal;
*μ_TL_*:	Poisson’s ratio for transverse–longitudinal;
*G_LR_*:	Shear modulus in the longitudinal–radial plane;
*G_LT_*:	Shear modulus in the longitudinal–transverse plane;
*G_RT_*:	Shear modulus in the radial–transverse plane.
